# Decoding the Liver–Blood Partitioning of Neonicotinoid Insecticides: Evidence from Paired Human Liver and Blood Biomonitoring

**DOI:** 10.3390/toxics14030237

**Published:** 2026-03-10

**Authors:** Jiaqi Shao, Tingna Chen, Yihan Li, Wenfei Yu, Hangbiao Jin, Qinghua Zhou, Yuanchen Chen

**Affiliations:** 1 Zhejiang Key Laboratory of Clean Energy Conversion and Utilization, Science and Education Integration College of Energy and Carbon Neutralization, Zhejiang University of Technology, Hangzhou 310014, China; 13905800026@139.com (J.S.); hangbiao@zjut.edu.cn (H.J.); 2 State Key Laboratory of Green Chemical Synthesis and Conversion, Science and Education Integration College of Energy and Carbon Neutralization, Zhejiang University of Technology, Hangzhou 310014, China; 3 Key Laboratory of Microbial Technology for Industrial Pollution Control of Zhejiang Province, College of Environment, Zhejiang University of Technology, Hangzhou 310014, China; 18950586027@163.com (T.C.); 18403880567@163.com (Y.L.); 2112127040@zjut.edu.cn (W.Y.); qhzhou@zjut.edu.cn (Q.Z.)

**Keywords:** neonicotinoid insecticides, human biomonitoring, liver–blood partitioning, target-organ exposure, internal distribution

## Abstract

Neonicotinoids (NEOs) are among the most widely used insecticides worldwide, and their increasing detection in environmental and human matrices has raised concerns about chronic exposure and potential health risks. However, human data on target-organ burdens and liver–blood partitioning of NEOs remain unclear. Here, we quantified nine NEOs in paired liver tissue and whole-blood samples from 234 individuals to characterize internal distribution patterns and liver–blood partitioning of NEOs in humans. Samples included both liver cancer patients and non-liver cancer individuals, enabling exploratory evaluation of disease-related differences. At least one NEO was detected in 84.6% of blood samples and 87.2% of liver samples, with median concentrations ranging from 0.15–3.52 ng/mL in blood and 0.39–10.99 ng/g in liver, respectively. Dinotefuran was the most abundant compound in both matrices, accounting for 43.9% of total NEOs in blood and 25.8% in liver, indicating substantial matrix-specific compositional differences. Blood-to-liver partition ratios (R_B/L) varied substantially among compounds and showed a significant inverse association with logKow (*p* = 0.026), suggesting physicochemical property-dependent partitioning. R_B/L values were generally lower in liver cancer patients, indicating a relative shift toward hepatic accumulation. In exploratory logistic regression analyses, hepatic concentrations of acetamiprid, dinotefuran, imidaclothiz, and thiamethoxam remained statistically associated with liver cancer status after adjustment for covariates. Overall, these findings highlight the importance of tissue-specific biomonitoring and internal partitioning for interpreting human exposure to NEOs and for reducing exposure misclassification when evaluating liver-related health outcomes.

## 1. Introduction

Neonicotinoid insecticides (NEOs) are systemic insecticides with nicotine-like structures and are widely used in agriculture and public health vector control due to their high efficacy and broad-spectrum activity [1,2,3,4]. With their extensive and long-term use, NEOs and their metabolites have been increasingly detected in environmental media and human biological samples, including surface waters, drinking water, urine, blood, and serum [5,6,7,8]. This widespread occurrence has raised ongoing concerns about chronic background exposure in the general population [3,9,10].

To date, most population-based biomonitoring studies have relied on easily accessible biological matrices—most commonly urine, serum, or whole blood—to estimate internal exposure to NEOs [5,6,11,12,13,14]. However, these matrices primarily reflect relatively recent intake and circulating levels and may not adequately capture target-organ burdens [15]. As a result, reliance on a single circulating matrix may lead to exposure misclassification when evaluating organ-specific health effects [16].

The liver plays a central role in xenobiotic uptake, biotransformation, and clearance and is a major target organ for the toxic effects of many environmental contaminants [17]. For several classes of pollutants, including per- and polyfluoroalkyl substances (PFAS), persistent organic pollutants (POPs), and metals, paired liver–blood measurements in humans have demonstrated pronounced compound-specific partitioning and substantial divergence between circulating levels and hepatic burdens [18,19,20]. These studies collectively indicate that liver–blood partitioning is governed by physicochemical properties and protein-binding characteristics, and that blood-based metrics alone may underestimate target-organ exposure [3,21].

In contrast, direct evidence on liver–blood partitioning of neonicotinoid insecticides in humans is still lacking. Although experimental studies have demonstrated hepatic uptake, distribution, and metabolism of NEOs [22,23,24,25,26,27], human data have been largely limited to single-matrix biomonitoring studies using urine or blood [11,12,13,14,28]. Whether blood-measured NEO concentrations reflect true hepatic burdens—and whether individual NEOs exhibit compound-specific partitioning patterns in humans—therefore remains unresolved.

In parallel, increasing attention has been directed toward potential liver-related health effects of NEO exposure. Toxicological studies suggest that several NEOs can induce oxidative stress, metabolic disruption, and inflammatory responses in hepatic tissue [22,23,24]. Population-level evidence has also linked NEO exposure to alterations in liver function indicators and metabolic outcomes [29]. However, without target-organ-specific exposure measurements, interpretation of such associations remains challenging, and the compounds most relevant to hepatic burden and liver-related outcomes may be misidentified [30].

Accordingly, the primary objective of this study was to establish a paired liver–blood biomonitoring framework for neonicotinoid insecticides in humans and to characterize their internal distribution and partitioning behavior. Using paired whole-blood and liver tissue samples from 234 individuals, we quantified nine individual NEOs (imidacloprid (IMI), acetamiprid (ACE), thiamethoxam (THIA), clothianidin (CLO), thiacloprid (THI), flonicamid (FLO), nitenpyram (NIT), imidaclothiz (IMID), and dinotefuran (DIN)), examined detection frequencies, concentration levels, and compositional differences across matrices, and calculated blood-to-liver partition ratios (R_B/L) to evaluate physicochemical determinants of internal distribution [30,31,32]. In addition, we explored whether liver–blood partitioning patterns and hepatic burdens differed between liver cancer patients and non-liver cancer individuals, providing organ-oriented context for interpreting epidemiological associations [33,34,35,36].

By explicitly integrating target-organ measurements with circulating biomarkers, this study aims to improve understanding of NEO internal disposition and to strengthen the interpretative framework for evaluating liver-related health outcomes associated with neonicotinoid exposure [16,30].

## 2. Methods and Materials

### 2.1. Study Population and Sample Collection

Between 2019 and 2021, we collected 234 paired liver tissue and whole-blood samples at the First Affiliated Hospital, Zhejiang University School of Medicine. The cohort included liver cancer patients (*n* = 125) and non-liver cancer participants (*n* = 109). To better represent contaminant occurrence in non-diseased hepatic tissue, normal liver tissue rather than tumor tissue was collected in this study. All participants provided written informed consent, and the study protocol was approved by the Clinical Research Ethics Committee of the First Affiliated Hospital, Zhejiang University School of Medicine. Demographic information was extracted from hospital medical records, including sex, age, body weight, height, and residential address (age groups: <40, 40–50, 51–60, and >60 years). The age of the participants ranged from 16 to 86 years; detailed sample size information is provided in Table S1. Disease status (liver cancer vs non-liver cancer) was considered in the subsequent analyses.

**Figure 1:**
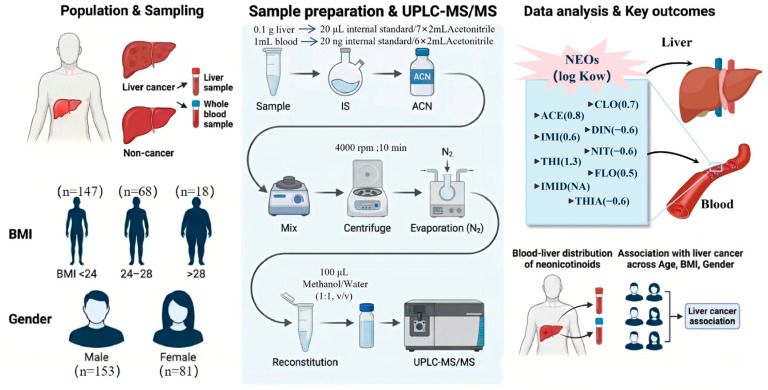
Study workflow and distributions of neonicotinoid insecticides (NEOs) in paired whole-blood and liver tissue samples.

Liver tissue samples were collected immediately after hepatic resection and placed into polypropylene (PP) tubes, then temporarily stored on dry ice. At the same time, 10–15 mL of paired whole blood was collected into EDTA-containing BD Vacutainer^®^ tubes (Becton, Dickinson and Company, Franklin Lakes, NJ, USA). We selected whole blood as the circulating matrix because it includes both the plasma and cellular fractions (primarily red blood cells), and thus can better reflect total circulating burdens for compounds that partition into blood cells. For NEOs, compound-specific partitioning between plasma and red blood cells has been reported in humans, with THIA showing predominant presence in red blood cells while several other NEOs (e.g., IMI and DIN) are mainly detected in plasma. Accordingly, whole-blood measurements may reduce matrix-driven bias for compounds with non-negligible cellular partitioning, but the resulting concentrations may not be directly comparable to values reported in serum- or plasma-based biomonitoring studies. Field blanks were prepared during sampling using ultrapure water (5.0 mL, *n* = 3). All samples were subsequently stored at −80 °C until analysis. An overview of the methodological workflow is illustrated in Figure 1.

### 2.2. Sample Preparation

For liver tissue samples, 0.1 g of freeze-dried liver tissue was ground into a fine powder and spiked with 20 μL of an isotopically labeled internal standard mixture (10 ppm), including ACE-d_3_, IMI-d_4_, CLO-d_3_, DIN-d_3_ and THIA-d_3_. Subsequently, 7 mL of acetonitrile was added as the extraction solvent. The mixture was vortexed for 2 min, shaken for 30 min, and ultrasonicated for 30 min, followed by centrifugation at 4000 rpm for 10 min. The supernatant was collected into a new 15 mL centrifuge tube. The residue was re-extracted with an additional 7 mL of acetonitrile under the same conditions, and the two supernatants were combined and evaporated to dryness under a gentle nitrogen stream. The residue was reconstituted with 100 μL of methanol/water (1:1, *v*/*v*), centrifuged at 13,000 rpm for 8 min, and the supernatant was transferred to amber autosampler vials for UPLC–MS/MS analysis.

The extraction procedure for whole-blood samples was similar to that for liver tissue, with minor adjustments in sample amount, solvent volume, and internal standard addition. Briefly, after thawing, 1 mL of whole blood was transferred into a 15 mL centrifuge tube and spiked with 200 ng of the isotopically labeled internal standard mixture (ACE-d_3_, IMI-d_4_, CLO-d_3_, DIN-d_3_, and THIA-d_3_). Then, 6 mL of acetonitrile was added for extraction. The samples were processed using the same vortexing, shaking, and ultrasonication conditions, followed by centrifugation at 4000 rpm for 10 min. The supernatant was collected, and the residue was re-extracted with an additional 6 mL of acetonitrile. The combined supernatants were evaporated to dryness under nitrogen, reconstituted with 100 μL of methanol/water (1:1, *v*/*v*), centrifuged at 13,000 rpm for 8 min, and the final supernatant was transferred to amber autosampler vials for instrumental analysis.

### 2.3. Instrumental Analysis

Quantification of nine target compounds—ACE, IMI, CLO, THIA, FLO, THI, NIT, DIN, and IMID—was performed using ultra-performance liquid chromatography coupled with tandem triple quadrupole mass spectrometry (UPLC–MS/MS; Xevo TQ-S Waters Corporation, Milford, MA, USA). Detailed information on the instrumental parameters can be found in Supporting Information.

### 2.4. Quality Assurance and Quality Control

During sample analysis, one acetonitrile blank was inserted after every eight samples to minimize potential carryover and cross-contamination between consecutive injections. The limits of detection (LOD) and limits of quantification (LOQ) for the nine neonicotinoid insecticides were defined as concentrations corresponding to signal-to-noise ratios (SNRs) of 3 and 10, respectively. LODs and LOQs were determined based on three independent replicate measurements, and the mean values were used.

For the nine neonicotinoid insecticides, The LOD ranges in liver tissue and whole blood were 0.009–0.027 ng/g and 0.009–0.021 ng/mL, respectively, while the corresponding LOQ ranges were 0.027–0.083 ng/g and 0.026–0.064 ng/mL.

In addition, recovery experiments were conducted by spiking randomly selected liver and blood samples with known concentrations of mixed standard solutions and isotopically labeled internal standards, followed by analysis under the same sample preparation and instrumental conditions. The recoveries of neonicotinoid insecticides ranged from 74.1% (THIA) to 112% (DIN) in liver samples and from 85.6% (THI) to 105% (ACE) in blood samples. All analyte recoveries met analytical quality requirements, indicating that the sample preparation procedures and UPLC–MS/MS conditions used in this study were appropriate and reliable.

### 2.5. Calculation of Blood-to-Liver Partition Ratio

The blood-to-liver partition ratio (R_B/L) was calculated to characterize the relative distribution of individual NEOs between paired whole blood and liver tissue as follows:

$$R\_B/L=\frac{C_{blood}}{C_{liver}}$$

where $C_{blood}$ and $C_{liver}$ represent the concentrations of individual NEOs in paired whole blood (ng/mL) and liver (ng/g), respectively. R_B/L values were calculated only when both blood and liver concentrations exceeded three times the LOD to reduce uncertainty near the detection limit.

### 2.6. Statistical Analysis

All statistical analyses were conducted using Python (v3.9). Data processing and management were performed using pandas (v1.3.5) and NumPy (1.21.6). Nonparametric tests and correlation analyses were implemented using SciPy (v1.7.3; scipy.stats). Logistic regression models were fitted using statsmodels (v0.13.2; Logit). Figures were generated using matplotlib (v3.4.3) and seaborn (v0.11.2). Continuous variables were assessed for distributional characteristics. Because NEO concentrations were right-skewed, nonparametric methods were applied. Differences between two independent groups were evaluated using the Mann–Whitney U test, and comparisons among more than two groups were assessed using the Kruskal–Wallis test. Correlations between continuous variables were assessed using Spearman’s rank correlation coefficient (ρ). Concentrations below the LOD were treated as non-detects and substituted with LOD/2 prior to subsequent analyses. ∑NEOs were calculated within each matrix as the sum of individual compounds after LOD substitution. For logistic regression analyses, NEO concentrations were entered as continuous predictors after natural log-transformation [ln(x)] to reduce right-skewness, limit the influence of extreme values, and improve model stability. Binary logistic regression was used to evaluate associations between NEO concentrations and liver cancer status, reporting odds ratios (ORs) with 95% confidence intervals (CIs). Both crude and multivariable-adjusted models were fitted; adjusted models included age, sex, and body mass index (BMI) as covariates. Values substituted as LOD/2 were included prior to ln-transformation. The R_B/L was calculated only when both liver and blood concentrations exceeded three times the LOD to reduce uncertainty near the detection limit; observations not meeting this criterion were treated as missing for partitioning analyses. All statistical tests were two-sided, and *p* < 0.05 was considered statistically significant. Where applicable, multiple-comparison adjustment was applied as specified in the corresponding analyses.

## 3. Result and Discussion

### 3.1. Levels and Composition of Neonicotinoids in Whole Blood and Liver

Paired whole-blood and liver tissue samples from 234 participants were analyzed. FLO was largely undetected (<LOD in most samples) and is therefore shown in figures and tables for completeness only; FLO was not included in inferential analyses and was not used to support interpretive conclusions throughout the Results and Discussion. In whole blood, at least one NEO was detected in 84.6% of samples. The mean and median ∑NEOs concentrations were 6.52 ± 4.44 ng/mL and 6.34 ng/mL, respectively, with concentrations ranging from non-detectable levels to 19.81 ng/mL (Table 1).

**Figure 2:**
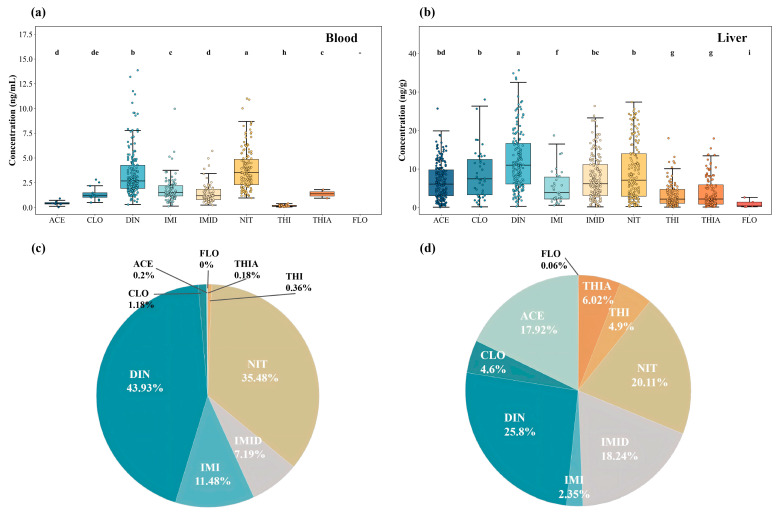
Concentrations and compositional profiles of nine neonicotinoid insecticides (NEOs) in paired whole-blood and liver tissue samples. (**a**) Whole-blood concentrations. (**b**) Liver tissue concentrations. (**c**,**d**) Mean compositional contributions of individual NEOs to ∑9NEOs in whole blood and liver tissue, respectively. FLO was largely <LOD and is shown for completeness only; it was not used for inferential interpretation. Different letters indicate significant differences among compounds in each panel, whereas shared letters indicate no significant difference (*p* < 0.05).

At the compound-specific level, the exposure composition in whole blood was highly uneven (Figure 2a,c). DIN and NIT dominated the mixture, accounting for 43.93% and 35.48% of ∑NEOs, respectively. DIN exhibited the highest detection frequency (84.6%) with a median concentration of 2.68 ng/mL. NIT was detected in 59.4% of samples and showed a median concentration of 3.52 ng/mL, contributing disproportionately among individuals with higher overall blood burdens. IMI and IMID contributed at intermediate levels (11.48% and 7.19%), with detection frequencies of 40.6% and 32.1% and median concentrations of 1.52 ng/mL and 1.19 ng/mL, respectively. The remaining compounds (ACE, CLO, THI, and THIA) were generally detected at lower frequencies or concentrations, and FLO was not detected in any whole-blood sample (Figure 2a).

Compared with previous biomonitoring studies, whole-blood concentrations of several high-frequency compounds were notably elevated. Earlier studies conducted in Guangdong Province reported mean DIN concentrations of 0.56 ng/mL and 0.95 ng/mL among students and individuals with obesity, respectively [12,37,38], whereas mean DIN concentrations in both liver cancer and non-liver cancer populations in the present study exceeded 3 ng/mL. Similarly, the mean IMI concentration in whole blood (1.8–2.0 ng/mL) was substantially higher than the previously reported level of approximately 0.21 ng/mL. These interstudy differences may reflect regional variation in crop structure and pesticide-use patterns, as well as differences in study populations, sampling periods, and analytical protocols [38,39,40].

In contrast to whole blood, neonicotinoid residues were substantially elevated in liver tissue. The mean hepatic concentration of ∑NEOs was 32.14 ± 19.61 ng/g, with a median of 32.69 ng/g, representing an approximately fivefold increase relative to whole-blood levels and a broader interindividual distribution. Hepatic ∑NEOs concentrations ranged from non-detectable levels (<LOD) to 88.45 ng/g (Table 1).

The compositional profile of neonicotinoids in liver tissue differed markedly from that in whole blood (Figure 2b,c). DIN, NIT, and IMID constituted the major contributors, accounting for 25.8%, 20.1%, and 18.2% of ∑NEOs, respectively. Notably, the contribution of ACE increased to approximately 18.0% in liver tissue, compared with only 3.0% in whole blood (Figure 2c). As illustrated in Figure 2d, the mean compositional structure of ∑NEOs in liver tissue was characterized by a more even distribution across multiple compounds than that observed in whole blood, with ACE, IMID, and THIA contributing substantially to the overall hepatic burden. DIN, NIT, and IMID also maintained high detection frequencies and concentration levels in liver tissue, with median concentrations of 10.99, 7.10, and 6.15 ng/g, respectively (Figure 2b).

Stratified analysis showed higher hepatic burdens among liver cancer patients than among non-liver cancer individuals. Notably, this comparison is cross-sectional and case–control in nature; therefore, the observed differences should be interpreted as associations rather than evidence of causation. The median hepatic concentration of ∑NEOs in the liver cancer group was 37.97 ng/g, exceeding that observed in the non-liver cancer group (32.53 ng/g). In liver cancer patients, detection frequencies of ACE, IMID, DIN, and NIT all exceeded 70%, with ACE detected in 89.6% of samples, substantially higher than the corresponding frequency in non-liver cancer individuals (77.1%) (Figure S1). As summarized in Figure S1, between-group contrasts were more evident in liver tissue than in whole blood, supporting the emphasis on hepatic burdens when interpreting liver cancer-related patterns. Because liver cancer may alter hepatic metabolism, transport processes, and blood flow, and may also involve treatment-related changes, disease-related shifts in liver–blood partitioning could contribute to the observed hepatic patterns; reverse causality cannot be excluded.

Previous studies on neonicotinoids in liver tissue have predominantly relied on animal exposure experiments, and population-level human evidence remains limited [16,25,26,27]. Here, we provide a systematic characterization of neonicotinoid residue levels and compositional profiles in human liver tissue based on paired biomonitoring. Given the liver’s central role in xenobiotic uptake, biotransformation, and clearance, and the fact that blood represents a dynamic circulating compartment, concurrent detection in both matrices is compatible with the presence of detectable parent compounds and/or metabolites in vivo and with measurable hepatic tissue burdens [16,41,42].

**Figure 3:**
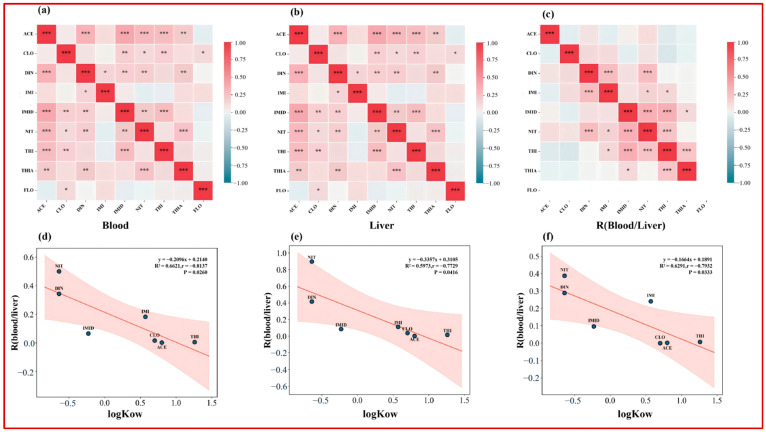
Co-exposure correlation structure of neonicotinoid insecticides (NEOs) in paired whole-blood and liver tissue samples, and associations between liver–blood partitioning and hydrophobicity. (**a**–**c**) Spearman correlation heatmaps for NEO concentrations in whole blood (**a**), liver tissue (**b**), and individual blood-to-liver partition ratios (R_B/L) (**c**). Colors indicate Spearman’s correlation coefficients (ρ; red, positive; blue, negative), and asterisks denote statistical significance (* *p* < 0.05, ** *p* < 0.01, *** *p* < 0.001). (**d**–**f**) Scatterplots showing relationships between logKow and liver–blood partitioning metrics across compounds, with linear regression fits based on blood concentrations (**d**), liver concentrations (**e**), and R_B/L values (**f**). Solid lines represent fitted regression lines, shaded areas indicate 95% confidence intervals, and points denote individual compounds (abbreviations shown). Regression equations, R^2^ values, and *p* values are reported within each panel. FLO was largely <LOD and is shown for completeness only; it was not used for inferential interpretation.

To further characterize the mixed-exposure structure, Spearman correlation analyses were conducted among individual neonicotinoids in whole blood and liver tissue (Figure 3a,b). In whole blood, significant positive correlations were observed for a limited number of compound pairs, mainly involving high-frequency analytes (e.g., DIN–IMI, DIN–THI, IMI–NIT, and IMI–THI). In liver tissue, correlations were more pervasive, with significant co-variation observed across multiple compound pairs involving ACE, DIN, IMID, NIT, THI, and THIA (*p* < 0.01). Overall, the broader correlation structure observed in liver tissue suggests that hepatic measurements integrate co-exposure signals while also reflecting compound-specific uptake, retention, and metabolism, consistent with previous human biomonitoring studies reporting correlated NEO profiles across biological matrices [12,28,41,43]. Taken together, the case–control differences and the subsequent hepatic association signals should be viewed as exploratory and hypothesis-generating. A primary implication is that reliance on blood-only biomarkers may lead to exposure misclassification when evaluating liver-related outcomes, underscoring the value of tissue-specific biomonitoring and internal partitioning information.

### 3.2. Liver–Blood Partitioning Characteristics Based on Population Biomonitoring

Previous studies, largely based on animal models or in vitro systems, have demonstrated that NEOs can enter the liver and undergo hepatic biotransformation [41]. Their internal distribution is therefore influenced not only by external exposure levels but also by molecular physicochemical properties and hepatic transport and metabolic processes [23,42,44,45]. Using paired whole-blood and liver tissue samples from the same individuals, we calculated blood-to-liver partition ratios (R_B/L) for individual NEOs to characterize their relative distribution between the circulating compartment and hepatic tissue.

Across the overall study population, R_B/L values varied substantially among compounds and were significantly negatively correlated with logKow (*p* = 0.026; Figure 2d). Compounds with lower hydrophobicity, such as DIN and NIT, exhibited higher median R_B/L values (approximately 0.5 and 1.5, respectively), indicating a relatively greater presence in whole blood [31]. In contrast, more hydrophobic compounds, including THI and ACE, showed markedly lower R_B/L values (approximately 0.09 and 0.07), reflecting a greater relative partitioning into liver tissue [32]. This compound-specific pattern is consistent with physicochemical property-dependent distribution, whereby increasing hydrophobicity favors association with lipid-rich phases and macromolecular binding within hepatic tissue, resulting in lower blood-to-liver ratios [23,45].

Liver–blood partitioning is unlikely to be determined by hydrophobicity alone, but rather reflects the combined influence of multiple processes, including membrane/lipid partitioning, binding to plasma and tissue macromolecules (i.e., the unbound fraction), ionization behavior, and transporter-mediated uptake and efflux [46,47]. Within certain chemical spaces, logKow may act as a proxy for factors that co-vary with lipophilicity [48], such as protein binding or hepatocellular binding capacity, and physicochemical properties including lipophilicity have been linked to interactions with hepatic uptake and efflux transporters, such as organic anion transporting polypeptides (OATPs) [49] and organic cation transporter 1 (OCT1) [50]. However, evidence for some neonicotinoids suggests that plasma protein binding does not systematically track lipophilicity, indicating that additional determinants beyond logKow contribute to tissue distribution. Because the present study did not directly measure plasma protein binding, tissue binding, transporter involvement, or intrahepatic metabolic profiles, the relative contributions of these mechanisms cannot be resolved. Therefore, the observed relationship between logKow and the liver-to-blood partition ratio should be interpreted as a descriptive physicochemical association rather than a causal mechanism, and as a hypothesis-generating basis for future mechanistic investigations.

Stratified analyses further showed that R_B/L values were generally lower in the liver cancer population than in non-liver cancer individuals (Figure S2). Consistently, the overall distribution of ΣNEOs exhibited a modest shift toward lower R_B/L in liver cancer patients (mean ± SD: 0.237 ± 0.234) compared with non-liver cancer individuals (0.287 ± 0.301), suggesting relatively greater hepatic enrichment. For DIN, the median R_B/L in liver cancer patients was 0.223 (IQR: 0.129–0.394), which was significantly lower than that observed in non-liver cancer individuals (median: 0.295; IQR: 0.188–0.714; Wilcoxon *p* = 0.009). NIT displayed a similar directional difference, with a lower median R_B/L in the liver cancer group (0.464) compared with the non-liver cancer group (0.646). Together, these results indicate an altered liver–blood partitioning pattern associated with liver cancer status, characterized by relatively lower blood-to-liver ratios and higher proportional hepatic burdens [28,36].

To explore whether shared determinants may underlie these partitioning patterns, we further examined the correlation structure among R_B/L values of individual NEOs using Spearman correlation analysis (Figure 3c). Several compounds exhibited significant positive correlations in their R_B/L values, suggesting partially shared factors influencing liver–blood distribution across NEOs. Overall, paired biomonitoring data support logKow-related liver–blood partitioning behavior of NEOs in humans and consistently indicate lower R_B/L values in liver cancer patients than in non-liver cancer individuals.

### 3.3. Sex- and BMI-Stratified Patterns of Neonicotinoid Burden

Using paired liver tissue and whole-blood samples from the same individuals, we examined sex- and BMI-stratified patterns of neonicotinoid burden. Sex differences were evaluated using the Mann–Whitney U test. BMI was analyzed both as a categorical variable (<24, 24–28, ≥28; Kruskal–Wallis test) and as a continuous variable using Spearman correlation analysis.

**Figure 4:**
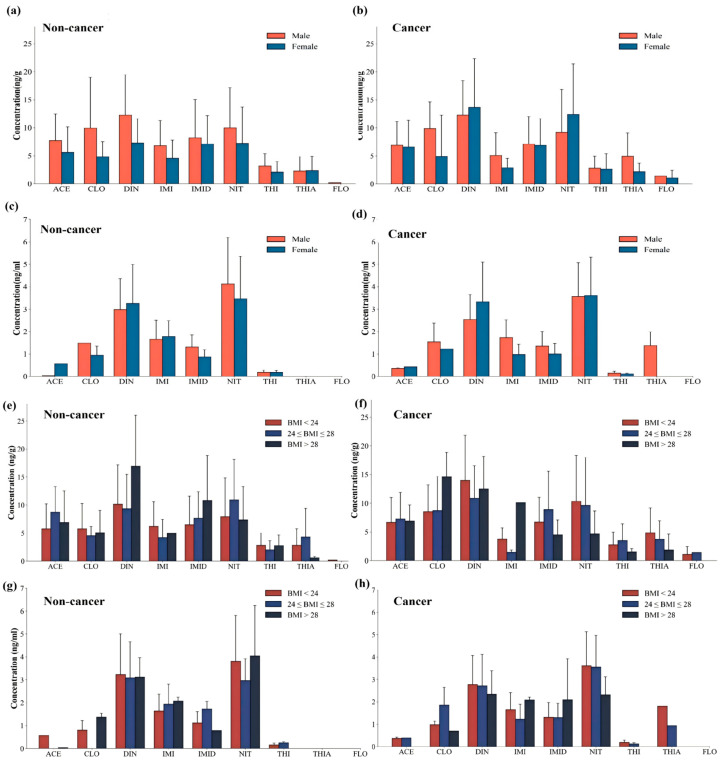
Neonicotinoid insecticide concentrations in non-liver cancer and liver cancer populations stratified by sex and BMI. (**a**,**b**) Hepatic concentrations of nine neonicotinoids in non-liver cancer (**a**) and liver cancer (**b**) individuals, stratified by sex. (**c**,**d**) Whole-blood concentrations of the same neonicotinoids in non-liver cancer (**c**) and liver cancer (**d**) individuals. (**e**,**f**) Hepatic concentrations of nine neonicotinoids in non-liver cancer (**e**) and liver cancer (**f**) individuals, stratified by BMI category. (**g**,**h**) Whole-blood concentrations of nine neonicotinoids in non-liver cancer (**g**) and liver cancer (**h**) individuals, stratified by BMI category. Data are presented as mean ± standard error. FLO was largely <LOD and is shown for completeness only; it was not used for inferential interpretation.

In liver tissue, sex differences were not observed for most analytes in the liver cancer population. However, among detected samples, THIA concentrations were significantly higher in males than in females (median: 3.08 vs. 1.85 ng/g, *p* = 0.028; Figure 4a,b). In the non-liver cancer population, males exhibited higher hepatic burdens for certain compounds: ACE concentrations were significantly higher in males than in females (median: 7.06 vs. 5.07 ng/g, *p* = 0.032; Figure 4a,b), and ∑NEOs were also elevated in males (38.10 vs. 30.66 ng/g, *p* = 0.027).

In whole blood, no significant sex differences were observed in the non-liver cancer population (*p* > 0.05). In contrast, sex-related differences were more evident in the liver cancer population. Among detected samples, males showed higher median IMI concentrations than females (1.66 vs. 0.91 ng/mL, *p* = 0.004; Figure 4c,d), and ∑NEOs were also higher in males (7.52 vs. 5.85 ng/mL, *p* = 0.045).

Sex-related differences in internal exposure have been reported inconsistently across previous neonicotinoid biomonitoring studies. Male-dominant patterns similar to those observed here have been reported in serum-based studies among elderly populations in southern China [51], whereas other studies have reported higher levels in females or no significant sex differences [12,52]. Such heterogeneity may reflect differences in exposure behaviors (e.g., occupational or agricultural contact, diet, and lifestyle) [53,54], physiological and toxicokinetic factors (e.g., body size, blood volume, hepatic blood flow, and sex hormone–regulated expression of CYP enzymes and transporters) [55,56], as well as methodological factors such as matrix selection and handling of non-detects. Notably, sex differences in whole blood were more pronounced in liver cancer patients than in non-liver cancer individuals, suggesting that disease status may modify systemic physiology and liver–blood exchange, thereby accentuating sex-specific contrasts for certain high-frequency compounds [57]. The determinants of these patterns remain uncertain and warrant confirmation in larger cohorts.

In liver tissue, BMI was associated with the burden of specific neonicotinoids. In the non-liver cancer population, ∑NEOs was positively correlated with BMI (*p* = 0.015), with median ∑NEOs increasing across BMI categories. Similarly, NIT concentrations were positively correlated with BMI in the non-liver cancer group (*p* = 0.040; Figure 4e). In contrast, THIA showed a negative correlation with BMI in both populations (non-liver cancer: *p* = 0.032; liver cancer: *p* = 0.028; Figure 4e,f), with lower post-detection median concentrations observed in individuals with obesity.

Group comparisons further indicated significant differences in hepatic ACE concentrations across BMI categories in the non-liver cancer population (Kruskal–Wallis *p* = 0.048; Figure 4g). The highest median ACE concentration was observed in the 24–28 BMI group (8.31 ng/g, IQR: 5.78–12.23), compared with the <24 group (4.51 ng/g, IQR: 1.82–8.62) and the ≥28 group (5.89 ng/g, IQR: 3.43–8.81).

By contrast, BMI was not consistently associated with ∑NEOs in whole blood (*p* > 0.05). Overall, these findings indicate that BMI-related variation in neonicotinoid burden was more evident in liver tissue than in whole blood. This pattern is consistent with the possibility that BMI may preferentially influence hepatic burden through alterations in the metabolic milieu of the liver—such as lipid accumulation, inflammatory status, intrahepatic blood flow, and transport processes—making such associations more detectable at the tissue level, while relationships in circulating blood appear less stable [58,59].

### 3.4. Liver Tissue-Based Associations Between Neonicotinoid Burden and Liver Cancer Status

To examine differences in NEO burden between liver cancer and non-liver cancer individuals, liver cancer status was specified as the outcome, and logistic regression models were constructed using quantitative measurements from whole blood (ng/mL) and liver tissue (ng/g), respectively. Crude odds ratios (ORs) and multivariable-adjusted ORs with 95% confidence intervals (CIs) were estimated (Table 2).

In whole blood, concentrations of individual NEOs were numerically higher in the liver cancer group; however, no compound showed a statistically significant association with liver cancer status in either crude or adjusted models (*p* > 0.05). Thus, within the present dataset, blood-based NEO concentrations did not provide statistically significant evidence for discrimination between liver cancer and non-liver cancer individuals.

In contrast, analyses based on liver tissue indicated more pronounced differences in NEO burden by disease status. Overall hepatic ∑NEOs concentrations were higher in liver cancer patients than in non-liver cancer individuals, with statistically significant differences observed for ACE, DIN, IMID, and THIA (*p* < 0.05; Table 2). Consistently, logistic regression analyses showed statistically significant associations between hepatic concentrations of ACE (OR = 0.75, 95% CI: 0.61–0.91, *p* = 0.004), DIN (OR = 0.83, 95% CI: 0.73–0.95, *p* = 0.007), IMID (OR = 0.84, 95% CI: 0.72–0.97, *p* = 0.019), and THIA (OR = 0.80, 95% CI: 0.67–0.95, *p* = 0.013) and liver cancer status. After adjustment for sex and BMI, these associations remained statistically significant: ACE (OR = 0.79, 95% CI: 0.64–0.99, *p* = 0.037), DIN (OR = 0.81, 95% CI: 0.70–0.93, *p* = 0.004), IMID (OR = 0.84, 95% CI: 0.71–0.98, *p* = 0.030), and THIA (OR = 0.79, 95% CI: 0.65–0.96, *p* = 0.017) (Table 2).

Overall, several NEOs exhibited statistically significant associations with liver cancer status when evaluated using hepatic tissue concentrations, whereas corresponding blood-based measurements did not. These findings highlight the importance of target-organ measurements for distinguishing internal exposure patterns by disease status [60,61,62]. From a biological perspective, the liver is the primary site of xenobiotic uptake, metabolism, and clearance, and toxicological studies have shown that multiple NEOs can induce oxidative stress, inflammatory responses, and metabolic perturbations in hepatic tissue, accompanied by altered biomarkers or histopathological changes [63]. This target-organ context may partly explain why disease-related differences were more evident in liver tissue than in whole blood [64].

Nevertheless, these associations should be interpreted cautiously. Liver cancer has multifactorial etiologies, and residual confounding by co-exposures, diet, pre-existing liver conditions, and treatment-related factors cannot be excluded. In addition, reverse causality and disease-related alterations in hepatic metabolism, transport, and chemical partitioning may influence tissue concentrations, complicating causal interpretation. Future studies incorporating mixture-based analytical approaches and sensitivity analyses, together with detailed clinical information on liver function, disease stage, and treatment history, will be essential for strengthening inference regarding neonicotinoid exposure and liver-related health outcomes [65,66].

## 4. Conclusions

This study reports population-based paired liver–blood biomonitoring data for NEOs from 234 individuals, providing direct evidence on internal distribution and liver–blood partitioning in humans. NEOs were frequently detected in both matrices, and hepatic ΣNEOs were markedly higher than whole-blood levels, indicating that blood measurements alone may underestimate target-organ burdens. Dinotefuran predominated in both matrices, yet mixture profiles differed between blood and liver, highlighting clear matrix-specific heterogeneity. R_B/L varied widely across compounds and showed a significant inverse relationship with logKow, consistent with physicochemical property-dependent partitioning in vivo. R_B/L values were generally lower in liver cancer patients, reflecting a shift toward hepatic enrichment under pathological conditions. In exploratory logistic regression models, several hepatic NEOs were associated with liver cancer status after adjustment for age, sex, and BMI, whereas corresponding blood-based metrics showed no comparable associations. Given the cross-sectional case–control design, these results are hypothesis-generating rather than causal and underscore the risk of exposure misclassification when liver-related outcomes are evaluated using blood-only biomarkers. Future work should prioritize longitudinal designs with richer clinical characterization and integrate mixture-aware analyses with toxicokinetic frameworks to clarify determinants of liver–blood partitioning and strengthen causal inference.

## Figures and Tables

**Table 1:** Detection rates and concentration characteristics of nine target NEOs and ΣNEOs in paired whole-blood and liver tissue samples from liver cancer (*n* = 125) patients and non-liver cancer (*n* = 109) individuals. FLO was largely <LOD and is shown for completeness only; it was not used for inferential interpretation.

		ACE	CLO	DIN	IMI	IMID	NIT	THI	THIA	FLO	ΣNEOs
Blood	Liver cancer (*n* = 125)										
	DRs	4.0%	7.2%	88.8%	39.2%	37.6%	61.6%	16.0%	1.6%	0	88.8%
	median	0.39	1.19	2.59	1.45	1.23	3.80	0.13	1.37	ND	7.07
	SD	0.24	0.79	2.44	0.97	0.89	2.15	0.09	0.61	ND	3.65
	mean	0.48	1.51	3.31	1.65	1.45	3.97	0.16	1.37	ND	7.60
	Non-cancer (*n* = 109)										
	DRs	1.8%	3.7%	79.8%	42.2%	25.7%	56.9%	11.9%	0	0	79.8%
	median	0.30	1.18	2.89	1.65	1.15	3.23	0.18	ND	ND	6.94
	SD	0.38	0.42	2.11	1.56	1.21	2.00	0.08	ND	ND	3.93
	mean	0.29	1.09	3.47	2.05	1.47	3.79	0.18	ND	ND	7.82
Liver	Liver cancer (*n* = 125)										
	DRs	89.6%	17.6%	73.6%	14.4%	83.2%	73.6%	50.4%	53.6%	3.2%	93.6%
	median	6.14	9.81	11.38	3.52	6.13	7.01	2.17	2.64	0.89	37.97
	SD	4.63	6.21	7.43	4.78	5.44	7.95	3.45	3.85	1.11	16.83
	mean	7.02	9.93	13.05	5.13	7.52	9.78	3.48	4.30	1.16	38.39
	Non-cancer (*n* = 109)										
	DRs	77.1%	16.51%	61.5%	13.8%	70.6%	65.1%	44.9%	44.0%	0.9%	79.8%
	median	6.05	4.87	9.99	4.97	6.45	7.19	2.52	1.28	0.19	32.53
	SD	4.71	6.66	7.83	3.88	5.97	6.91	2.74	4.18	0	15.46
	mean	6.68	7.10	11.04	5.61	7.65	8.62	3.04	3.43	0.19	34.80

Notes: DRs = detection rates; SD = standard deviation; ND = not detected (concentration below the limit of detection, LOD).

**Table 2:** In the studies conducted on the liver cancer population (*n* = 125) and the healthy population (*n* = 109), the probability of diagnosing liver cancer through the liver and blood concentrations of NEO.

Compounds	Crude OR	Crude *p*	Adjusted OR	Adjusted ^a^ *p*
	(95% CI)		(95% CI)	
Liver (*n* = 234)				
ACE	0.75(0.61–0.91)	0.004	0.79(0.64–0.99)	0.037
CLO	0.94(0.78–1.14)	0.549	0.97(0.80–1.19)	0.800
DIN	0.83(0.73–0.95)	0.007	0.81(0.70–0.93)	0.004
IMI	0.99(0.84–1.16)	0.913	0.96(0.81–1.15)	0.666
IMID	0.84(0.72–0.97)	0.019	0.84(0.71–0.98)	0.030
NIT	0.83(0.68–1.01)	0.061	0.86(0.70–1.06)	0.162
THI	0.96(0.89–1.04)	0.311	0.98(0.90–1.07)	0.638
THIA	0.80(0.67–0.95)	0.013	0.79(0.65–0.96)	0.017
∑NEOs	0.65(0.50–0.83)	0.006	0.68(0.52–0.88)	0.004
Blood (*n* = 234)				
ACE	0.17(0.01–5.08)	0.307	0.11(0.00–5.86)	0.274
CLO	0.64(0.34–1.22)	0.176	0.61(0.30–1.24)	0.171
DIN	0.87(0.70–1.09)	0.229	0.84(0.66–1.07)	0.157
IMI	1.06(0.92–1.22)	0.406	1.05(0.90–1.22)	0.548
IMID	0.79(0.62–1.01)	0.062	0.84(0.65–1.10)	0.212
NIT	0.88(0.65–1.18)	0.376	0.98(0.71–1.34)	0.880
THI	0.92(0.76–1.12)	0.424	0.91(0.74–1.13)	0.398
THIA	0.80(0.67–0.95)	1.000	0.00(0.00–inf)	1.000
∑NEOs	0.77(0.52–1.13)	0.311	0.79(0.52–1.19)	0.261

^a^ It is adjusted according to gender, age and BMI.

## Data Availability

The data supporting the conclusions of this article will be made available by the corresponding authors upon reasonable request.

## Supplementary Materials

The following supporting information can be downloaded at: https://www.mdpi.com/article/10.3390/toxics14030237/s1. Table S1. Demographic characteristics of the study participant; Table S2. Quantitative Ion Pairs of New Pyrethroid Insecticides and Standard Curve; Table S3. The chemical structures and physicochemical properties of the individual neonicotinoids; Table S4. The neonicotinoid residues in the human blood samples for different demographic groups; Table S5. The neonicotinoid residues in the human liver samples for different demographic groups; Figure S1. Cancer (**a**) and non-cancer (**b**) populations distributions of R(Blood/Liver); (**c**) Comparison of R(Blood/Liver) for ∑NEOs between groups; Figure S2. Comparison of the mean concentrations of NEOs in liver tissue (**a**) and whole blood (**b**) between the liver cancer group and the non-liver cancer group.

## Abbreviations

The following abbreviations are used in this manuscript:

NEOs	Neonicotinoids
R_B/L	Blood-to-liver partition ratios
IMI	Iimidacloprid
ACE	Acetamiprid
THIA	Thiamethoxam
CLO	Clothianidin
THI	Thiacloprid
FLO	Flonicamid
NIT	Nitenpyram
IMID	Imidaclothiz
DIN	Dinotefuran
PFAS	Polyfluoroalkyl substances
POPs	Persistent organic pollutants
ACE-d_3_	Acetamiprid-d_3_
IMI-d_4_	Imidacloprid-d_4_
CLO-d_3_	Clothianidin-d_3_
DIN-d_3_	Dinotefuran-d_3_
THIA-d_3_	Thiamethoxam-d_3_
LOD	The limits of detection
LOQ	The limits of quantification
SNRs	Signal-to-noise ratios
ORs	Odds ratios
CIs	Confidence intervals
BMI	Body mass index
OATPs	Organic anion transporting polypeptides
OCT1	Organic cation transporter 1

## References

[1] Jeschke P., Nauen R., Schindler M., Elbert A.J. (2011). Overview of the status and global strategy for neonicotinoids. J. Agric. Food Chem..

[2] Chen Y., Yan C., Sun Z., Wang Y., Tao S., Shen G., Xu T., Zhou P., Cao X., Wang F.J.E.S. (2021). Organochlorine pesticide ban facilitated reproductive recovery of Chinese striped hamsters. Environ. Sci. Technol..

[3] Hladik M.L., Main A.R., Goulson D. (2018). Environmental risks and challenges associated with neonicotinoid insecticides. Environ. Sci. Technol..

[4] Thompson D.A., Lehmler H.-J., Kolpin D.W., Hladik M.L., Vargo J.D., Schilling K.E., LeFevre G.H., Peeples T.L., Poch M.C., LaDuca L.E. (2020). A critical review on the potential impacts of neonicotinoid insecticide use: Current knowledge of environmental fate, toxicity, and implications for human health. Environ. Sci. Process. Impacts.

[5] Zhang D., Lu S. (2022). Human exposure to neonicotinoids and the associated health risks: A review. Environ. Int..

[6] Ospina M., Wong L.-Y., Baker S.E., Serafim A.B., Morales-Agudelo P., Calafat A.M. (2019). Exposure to neonicotinoid insecticides in the US general population: Data from the 2015–2016 national health and nutrition examination survey. Environ. Res..

[7] Klarich K.L., Pflug N.C., DeWald E.M., Hladik M.L., Kolpin D.W., Cwiertny D.M., LeFevre G.H., Letters T. (2017). Occurrence of neonicotinoid insecticides in finished drinking water and fate during drinking water treatment. Environ. Sci. Technol. Lett..

[8] Berens M.J., Capel P.D., Arnold W.A. (2021). Neonicotinoid insecticides in surface water, groundwater, and wastewater across land-use gradients and potential effects. Environ. Toxicol. Chem..

[9] Woodcock B.A., Isaac N.J., Bullock J.M., Roy D.B., Garthwaite D.G., Crowe A., Pywell R.F. (2016). Impacts of neonicotinoid use on long-term population changes in wild bees in England. Nat. Commun..

[10] Woodcock B.A., Bullock J.M., Shore R.F., Heard M.S., Pereira M.G., Redhead J., Ridding L., Dean H., Sleep D., Henrys P.J.S. (2017). Country-specific effects of neonicotinoid pesticides on honey bees and wild bees. Science.

[11] Wrobel S.A., Bury D., Hayen H., Koch H.M., Brüning T., Käfferlein H.U. (2022). Human metabolism and urinary excretion of seven neonicotinoids and neonicotinoid-like compounds after controlled oral dosages. Arch. Toxicol..

[12] Xu M., Zhang Z., Li Z., Kan S., Liu Z., Wang D., Liu Q., Zhang H. (2021). Profiles of neonicotinoid insecticides and characteristic metabolites in paired urine and blood samples: Partitioning between urine and blood and implications for human exposure. Sci. Total. Environ..

[13] Yao Y.-N., Song S., Huang Y., Kannan K., Sun H., Zhang T. (2023). Insights into free and conjugated forms of neonicotinoid insecticides in human serum and their association with oxidative stress. Environ. Heal..

[14] Chen Q., Zhang Y., Li J., Su G., Chen Q., Ding Z., Sun H. (2021). Serum concentrations of neonicotinoids, and their associations with lipid molecules of the general residents in Wuxi City, Eastern China. J. Hazard. Mater..

[15] Calafat A.M., Longnecker M.P., Koch H.M., Swan S.H., Hauser R., Goldman L.R., Lanphear B.P., Rudel R.A., Engel S.M., Teitelbaum S.L. (2015). Optimal exposure biomarkers for nonpersistent chemicals in environmental epidemiology. Environ. Heal. Perspect..

[16] Chen Y., Yu W., Zhang L., Cao L., Ling J., Liao K., Shen G., Du W., Chen K., Zhao M. (2023). First evidence of neonicotinoid insecticides in human bile and associated hepatotoxicity risk. J. Hazard. Mater..

[17] Gu X., Manautou J.E. (2012). Molecular mechanisms underlying chemical liver injury. Expert Rev. Mol. Med..

[18] Hong Y., Wang D., Liu Z., Chen Y., Wang Y., Li J. (2025). Decoding per-and polyfluoroalkyl substances (PFAS) in hepatocellular carcinoma: A multi-omics and computational toxicology approach. J. Transl. Med..

[19] Zani C., Gelatti U., Donato F., Capelli M., Portolani N., Bergonzi R., Apostoli P. (2013). Polychlorinated biphenyls in serum, liver and adipose tissue of subjects with hepatocellular carcinoma living in a highly polluted area. Chemosphere.

[20] Lee J., Fraser M., Philibert A., Saint-Amour D., Mergler D., Fillion M. (2025). Mercury concentrations in historic autopsies from Grassy Narrows First Nation. J. Neurol. Sci..

[21] Morrissey C.A., Mineau P., Devries J.H., Sanchez-Bayo F., Liess M., Cavallaro M.C., Liber K. (2015). Neonicotinoid contamination of global surface waters and associated risk to aquatic invertebrates: A review. Environ. Int..

[22] Wang X., Anadón A., Wu Q., Qiao F., Ares I., Martínez-Larrañaga M.-R., Yuan Z., Martínez M.-A. (2018). Mechanism of neonicotinoid toxicity: Impact on oxidative stress and metabolism. Annu. Rev. Pharmacol. Toxicol..

[23] Yan S., Meng Z., Tian S., Teng M., Yan J., Jia M., Li R., Zhou Z., Zhu W. (2020). Neonicotinoid insecticides exposure cause amino acid metabolism disorders, lipid accumulation and oxidative stress in ICR mice. Chemosphere.

[24] Zheng M., Qin Q., Zhou W., Liu Q., Zeng S., Xiao H., Bai Q., Gao J. (2020). Metabolic disturbance in hippocampus and liver of mice: A primary response to imidacloprid exposure. Sci. Rep..

[25] Hirano T., Ohno S., Ikenaka Y., Onaru K., Kubo S., Miyata Y., Maeda M., Mantani Y., Yokoyama T., Nimako C. (2024). Quantification of the tissue distribution and accumulation of the neonicotinoid pesticide clothianidin and its metabolites in maternal and fetal mice. Toxicol. Appl. Pharmacol..

[26] Duzguner V., Erdogan S. (2010). Acute oxidant and inflammatory effects of imidacloprid on the mammalian central nervous system and liver in rats. Pestic. Biochem. Physiol..

[27] Kapoor U., Srivastava M., Trivedi P., Garg V., Srivastava L. (2014). Disposition and acute toxicity of imidacloprid in female rats after single exposure. Food Chem. Toxicol..

[28] Zhang Q., Hu S., Dai W., Gu S., Ying Z., Wang R., Lu C. (2023). The partitioning and distribution of neonicotinoid insecticides in human blood. Environ. Pollut..

[29] Godbole A.M., Chen A., Vuong A.M. (2024). Associations between neonicotinoids and liver function measures in US adults: National health and nutrition examination survey 2015–2016. Environ. Epidemiol..

[30] Baumert B.O., Fischer F.C., Nielsen F., Grandjean P., Bartell S., Stratakis N., Walker D.I., Valvi D., Kohli R., Inge T. (2023). Paired liver: Plasma PFAS concentration ratios from adolescents in the Teen-LABS study and derivation of empirical and mass balance models to predict and explain liver PFAS accumulation. Environ. Sci. Technol..

[31] Poulin P., Theil F.P. (2000). A priori prediction of tissue: Plasma partition coefficients of drugs to facilitate the use of physiologically-based pharmacokinetic models in drug discovery. J. Pharm. Sci..

[32] Holt K., Ye M., Nagar S., Korzekwa K. (2019). Prediction of tissue-plasma partition coefficients using microsomal partitioning: Incorporation into physiologically based pharmacokinetic models and steady-state volume of distribution predictions. Drug Metab. Dispos..

[33] Barouki R., Samson M., Blanc E.B., Colombo M., Zucman-Rossi J., Lazaridis K.N., Miller G.W., Coumoul X. (2023). The exposome and liver disease-how environmental factors affect liver health. J. Hepatol..

[34] Beier J.I., Luo J., Vanderpuye C.-M., Brizendine P., Muddasani P., Bolatimi O., Heinig S.A., Ekuban F.A., Siddiqui H., Ekuban A. (2025). Environmental Pollutants, Occupational Exposures, and Liver Disease. Semin. Liver Dis..

[35] Wang X., Zhang L., Dong B. (2025). Molecular mechanisms in MASLD/MASH-related HCC. Hepatology.

[36] Billington S., Ray A.S., Salphati L., Xiao G., Chu X., Humphreys W.G., Liao M., Lee C.A., Mathias A., Hop C.E.C.A. (2018). Transporter expression in noncancerous and cancerous liver tissue from donors with hepatocellular carcinoma and chronic hepatitis C infection quantified by LC-MS/MS proteomics. Drug Metab. Dispos..

[37] Zhang M., Zhu J., Zheng P., Wei C., Li D., Wang Q., Zhang H. (2024). Neonicotinoid insecticides and their characteristic metabolites may induce high fasting blood glucose and obesity in human. Expo. Heal..

[38] Zhang T., Song S., Bai X., He Y., Zhang B., Gui M., Kannan K., Lu S., Huang Y., Sun H. (2019). A nationwide survey of urinary concentrations of neonicotinoid insecticides in China. Environ. Int..

[39] Yu W., Wu R., Zhang L., Pan Y., Ling J., Yang D., Qu J., Tao Z., Meng R., Shen Y. (2024). Identification of key factors affecting neonicotinoid residues in crops and risk of dietary exposure. Environ. Pollut..

[40] Hou J., Chen L., Wang J., Wang L., Han B., Li Y., Yu L., Liu W. (2025). Neonicotinoid metabolites in farmland surface soils in China based on multiple agricultural influencing factors: A national survey. J. Hazard. Mater..

[41] Li L., Liang H., Zhao T., Liu Y., Yan S., Zhu W. (2022). Differential effects of thiamethoxam and clothianidin exposure on their tissue distribution and chronic toxicity in mice. Chem. Interact..

[42] Taira K., Ikenaka Y., Bonmatin J.-M., Safer A. (2025). Human plasma protein bindings of neonicotinoid insecticides and metabolites. Sci. Rep..

[43] Thompson D.A., Kolpin D.W., Hladik M.L., Lehmler H.-J., Meppelink S.M., Poch M.C., Vargo J.D., Soupene V.A., Irfan N.M., Robinson M. (2023). Prevalence of neonicotinoid insecticides in paired private-well tap water and human urine samples in a region of intense agriculture overlying vulnerable aquifers in eastern Iowa. Chemosphere.

[44] Shi X., Dick R.A., Ford K.A., Casida J.E. (2009). Enzymes and inhibitors in neonicotinoid insecticide metabolism. J. Agric. Food Chem..

[45] Swenson T.L., Casida J.E. (2013). Aldehyde oxidase importance in vivo in xenobiotic metabolism: Imidacloprid nitroreduction in mice. Toxicol. Sci..

[46] Schmitt W. (2008). General approach for the calculation of tissue to plasma partition coefficients. Toxicol. Vitr..

[47] Rodgers T., Rowland M. (2006). Physiologically based pharmacokinetic modelling 2: Predicting the tissue distribution of acids, very weak bases, neutrals and zwitterions. J. Pharm. Sci..

[48] Zoghbi S.S., Anderson K.B., Jenko K.J., Luckenbaugh D.A., Innis R.B., Pike V.W. (2012). On quantitative relationships between drug-like compound lipophilicity and plasma free fraction in monkey and human. J. Pharm. Sci..

[49] Karlgren M., Vildhede A., Norinder U., Wisniewski J.R., Kimoto E., Lai Y., Haglund U., Artursson P. (2012). Classification of inhibitors of hepatic organic anion transporting polypeptides (OATPs): Influence of protein expression on drug–drug interactions. J. Med. Chem..

[50] Ahlin G., Karlsson J., Pedersen J.M., Gustavsson L., Larsson R., Matsson P., Norinder U., Bergström C.A.S., Artursson P. (2008). Structural requirements for drug inhibition of the liver specific human organic cation transport protein 1. J. Med. Chem..

[51] Zhang H., Zhu K., Du J., Ou M., Hou J., Wang D., Wang J., Zhang W., Sun G. (2022). Serum concentrations of neonicotinoids and their characteristic metabolites in elderly population from South China: Association with osteoporosis. Environ. Res..

[52] Song S., Zhang T., Huang Y., Zhang B., Guo Y., He Y., Huang X., Bai X.-Y., Kannan K. (2020). Urinary metabolites of neonicotinoid insecticides: Levels and recommendations for future biomonitoring studies in China. Environ. Sci. Technol..

[53] Manfo F.P.T., Nimako C., Nantia E.A., Suh C.F., Chenwi S.P., Cho-Ngwa F., Moundipa P.F., Nakayama S.M.M., Ishizuka M., Ikenaka Y. (2024). Exposure of male farmers and nonfarmers to neonicotinoid pesticides in the South-West and littoral regions of Cameroon: A comparative study. Environ. Toxicol. Chem..

[54] Tao Y., Dong F., Xu J., Phung D., Liu Q., Li R., Liu X., Wu X., He M., Zheng Y. (2019). Characteristics of neonicotinoid imidacloprid in urine following exposure of humans to orchards in China. Environ. Int..

[55] Waxman D.J., Holloway M.G. (2009). Sex differences in the expression of hepatic drug metabolizing enzymes. Mol. Pharmacol..

[56] Yang L., Li Y., Hong H., Chang C.-W., Guo L.-W., Lyn-Cook B., Shi L., Ning B. (2012). Sex differences in the expression of drug-metabolizing and transporter genes in human liver. J. Drug Metab. Toxicol..

[57] Wu J., LoRusso P.M., Matherly L.H., Li J. (2012). Implications of plasma protein binding for pharmacokinetics and pharmacodynamics of the γ-secretase inhibitor RO4929097. Clin. Cancer Res..

[58] Zhang T., Calvier E.A.M., Krekels E.H.J., Knibbe C.A.J. (2024). Impact of obesity on hepatic drug clearance: What are the influential variables?. AAPS J..

[59] Wang H., Yang D., Fang H., Han M., Tang C., Wu J., Chen Y., Jiang Q. (2020). Predictors, sources, and health risk of exposure to neonicotinoids in Chinese school children: A biomonitoring-based study. Environ. Int..

[60] Imagined M.C.T. (2017). Reverse causality in cardiovascular epidemiological research. Circulation.

[61] Chen H. (2025). Roles of organic anion transporting polypeptides in hepatocellular carcinoma. Front. Genet..

[62] Yan T., Lu L., Xie C., Chen J., Peng X., Zhu L., Wang Y., Li Q., Shi J., Zhou F. (2015). Severely impaired and dysregulated cytochrome P450 expression and activities in hepatocellular carcinoma: Implications for personalized treatment in patients. Mol. Cancer Ther..

[63] Wang Y., Zhang Y., Li W., Yang L., Guo B. (2019). Distribution, metabolism and hepatotoxicity of neonicotinoids in small farmland lizard and their effects on GH/IGF axis. Sci. Total. Environ..

[64] Qumsani A.T. (2024). Exploring the effects of imidacloprid on liver health and the microbiome in rats: A comprehensive study. Microorganisms.

[65] VanderWeele T.J., Ding P. (2017). Sensitivity analysis in observational research: Introducing the E-value. Ann. Intern. Med..

[66] Lipsitch M., Tchetgen E.T., Cohen T. (2010). Negative controls: A tool for detecting confounding and bias in observational studies. Epidemiology.

