# Supplementary material for: Decoding the Liver–Blood Partitioning of Neonicotinoid Insecticides: Evidence from Paired Human Liver and Blood Biomonitoring
Source: Toxics. 2026 Mar 10;14(3):237. doi: 10.3390/toxics14030237 (PMC13029847; doi:10.3390/toxics14030237)
Supplement: Supplementary file 1 [file toxics-14-00237-s001.zip › toxics-4162514-supplementary.pdf]

# SUPPORTING INFORMATION

# Decoding the Liver–Blood Partitioning of Neonicotinoid Insecticides: Evidence from Paired Human Liver and Blood Biomonitoring

**Jiaqi Shao [1,2], Tingna Chen [3], Yihan Li [3], Wenfei Yu [3], Hangbiao Jin [1,2], Qinghua Zhou [3] and Yuanchen Chen [1,2,*]**

[1] Zhejiang Key Laboratory of Clean Energy Conversion and Utilization, Science and Education Integration College of Energy and Carbon Neutralization, Zhejiang University of Technology, Hangzhou 310014, China; 13905800026@139.com (J.S.); hangbiao@zjut.edu.cn (H.J.)

[2] State Key Laboratory of Green Chemical Synthesis and Conversion, Science and Education Integration College of Energy and Carbon Neutralization, Zhejiang University of Technology, Hangzhou 310014, China

[3] Key Laboratory of Microbial Technology for Industrial Pollution Control of Zhejiang Province, College of Environment, Zhejiang University of Technology, Hangzhou 310014, China; 18950586027@163.com (T.C.); 18403880567@163.com (Y.L.); 2112127040@zjut.edu.cn (W.Y.); qhzhou@zjut.edu.cn (Q.Z.)

* Correspondence: chenyuanchen1988@zjut.edu.cn

**Instrumental Analysis** Quantification of nine target compounds—ACE, IMI, CLO, THIA, FLO, THI, NIT, DIN, and IMID—was performed using ultra-performance liquid chromatography coupled with tandem triple quadrupole mass spectrometry (UPLC–MS/MS; Waters Xevo TQ-S). Chromatographic separation was achieved on an ACQUITY UPLC BEH C18 column (2.1 mm × 50 mm, 1.7 μm) using gradient elution. The mobile phases consisted of methanol (A) and ultrapure water containing 0.01% acetic acid (B). The gradient program was as follows: 0–0.5 min, 5% A and 95% B; 1.0 min, 60% A and 40% B; 3.0 min, 80% A and 20% B; 4.0 min, 90% A and 10% B; 6.0 min, 90% A and 10% B; 6.1 min, 5% A and 95% B; and 9.0 min, 5% A and 95% B. The total run time for each sample was 9 min, with a flow rate of 0.2 mL/min and a column temperature of 35 °C.

Mass spectrometric detection was conducted using an electrospray ionization (ESI) source operated in positive ion mode. Multiple reaction monitoring (MRM) was employed for quantitative analysis of the target compounds as well as the isotopically labeled internal standards (ACE-$d_3$, DIN-$d_3$, IMI-$d_4$, CLO-$d_3$, and THIA-$d_3$). Detailed MRM transitions and optimized parameters for each analyte and internal standard are provided in Tables 2–4. Nitrogen was used as the nebulizing and desolvation gas, and argon was employed as the collision gas, with a constant gas flow rate of 1.0 mL/min. The ion source temperature was set to 300 °C, and the capillary and cone voltages were set to 2600 V and 48 V, respectively.

**Table S1. Demographic characteristics of the study participant**

| Characteristic | N (%) or mean ± SD |
|---|---|
| **Gender** | |
| Female | 81 (35%) |
| Male | 153 (65%) |
| **Age, years** | |
| Female | 54 ± 13 |
| Male | 57 ± 12 |
| < 40 years | 24 (10%) |
| 40–50 years | 42 (18%) |
| 51–60 years | 71 (30%) |
| > 60 years | 97 (41%) |
| **Weight (kg)** | |
| Female | 56 ± 7.8 |
| Male | 67 ± 10.4 |
| **Height (cm)** | |
| Female | 158 ± 4.7 |
| Male | 169 ± 5.4 |

**Table S2. Quantitative Ion Pairs of New Pyrethroid Insecticides and Standard Curve**

| Pesticides | Parent ion | Product ion | Standard Curve | $R^2$ |
|---|---|---|---|---|
| ACE | 223 | 126 | y=0.9478x+0.0034 | 0.9996 |
| CLO | 250 | 169 | y=1.0573x+0.0076 | 0.9996 |
| DIN | 203 | 129 | y=1.1843x+0.0057 | 0.9997 |
| NIT | 271 | 126 | y=0.0156x+0.00004 | 0.9997 |
| IMI | 256 | 209 | y=0.8964x+0.0022 | 0.9995 |
| THI | 253 | 126 | y=1.2975x+0.0042 | 0.9995 |
| THIA | 292 | 211 | y=0.4855x+0.0020 | 0.9998 |
| FLO | 230 | 203 | y=0.2565x+0.0341 | 0.9994 |
| IMID | 262 | 181 | y=1.8492x+0.0371 | 0.9994 |

**Table S3. The chemical structures and physicochemical properties of the individual neonicotinoids.**

| Compounds | CAS No. | Chemical structure | Molecular mass, g/mol | Solubility, g/L | Log $K_{ow}$ |
|---|---|---|---|---|---|
| Acetamiprid (ACE) | 135410-20-7 | 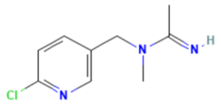 | 222.7 | 4.2 | 0.8 |
| Clothianidin (CLO) | 210880-92-5 | 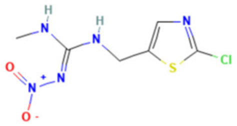 | 249.7 | 0.327 | 0.7 1 |
| Dinotefuran (DIN) | 165252-70-0 | 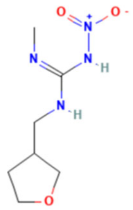 | 202.2 | 54.3 | -0.64 |
| Imidacloprid (IMI) | 138261-41-3 | 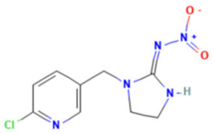 | 255.7 | 0.51 | 0.57 |
| Imidaclothiz (IMID) | 105843-36-5 | 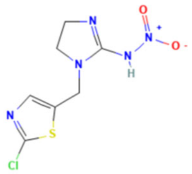 | 261.7 | 5 | NA |
| Nitenpyram (NIT) | 150824-47-8 | 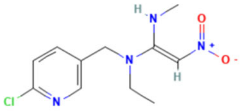 | 270.7 | 840 | -0.64 |
| Thiacloprid (THI) | 111988-49-9 | 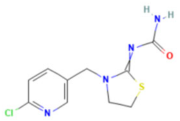 | 252.7 | 0.185 | 1.26 |

| | | | | | |
|---|---|---|---|---|---|
| Thiamethoxam (THIA) | 153719-23-4 | 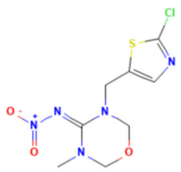 | 291 | 4.1 | -0.13 |
| Flonicamid (FLO) | 158062-67-0 | 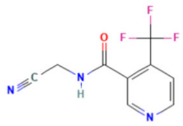 | 229.2 | 5.25 | 0.85 |

**Data source:** PubChem Open Chemistry Database, U.S. National Library of Medicine, National Institutes of Health (NIH). Available at: https://pubchem.ncbi.nlm.nih.gov/

**Note:** Log $K_{ow}$: n-octanol-water partition coefficient.

**Table S4. The neonicotinoid residues in the human blood samples for different demographic groups.**

| Characteristic | | | ACE | CLO | DIN | IMI | IMID | NIT | THI | THIA | FLO | ΣNEOs |
|---|---|---|---|---|---|---|---|---|---|---|---|---|
| **Detection frequency (DF, %)** | | | 3 | 5.6 | 84.6 | 40.6 | 32.1 | 59.4 | 14.1 | 0.9 | 0 | 84.6 |
| **Age，year** | **Sample size** | **level** | | | | | ng/mL | | | | | |
| 10-30 | 6 | median | <LOD | <LOD | 1.71 | <LOD | 1.99 | 3.69 | <LOD | <LOD | <LOD | 5.57 |
| | | mean±SD | <LOD | <LOD | 2.47±1.23 | <LOD | 3.10±NA | 1.32±0.94 | 3.76±0.44 | <LOD | <LOD | 6.63±3.38 |
| 30-60 | 126 | median | 0.36 | 1.21 | 2.67 | 1.62 | 1.3 | 3.48 | 0.15 | 1.8 | <LOD | 7.25 |
| | | mean±SD | 0.42±0.13 | 1.39±0.73 | 3.37±2.41 | <LOD | 1.82±0.90 | 1.61±1.07 | 3.78±2.08 | 0.17±0.10 | 1.80±NA | 7.83±3.91 |
| 60-90 | 102 | median | 0.41 | 1.12 | 2.72 | 1.49 | 1.12 | 3.52 | 0.15 | 0.94 | <LOD | 6.92 |
| | | mean±SD | 0.44±0.36 | 1.33±0.76 | 3.45±2.21 | <LOD | 1.84±1.69 | 1.22±0.90 | 4.05±2.17 | 0.16±0.08 | 0.94±NA | 7.60±3.62 |
| **BMI，kg/m2** | | | | | | | | | | | | |
| <24 | 147 | median | 0.43 | 1.06 | 2.64 | 1.51 | 1.16 | 3.53 | 0.15 | 1.8 | <LOD | 6.99 |
| | | mean±SD | 0.52±0.24 | 1.18±0.70 | 3.50±2.49 | <LOD | 1.75±0.96 | 1.32±0.88 | 3.91±2.04 | 0.17±0.09 | 1.80±NA | 7.76±3.71 |
| 24-28 | 68 | median | 0.39 | 1.7 | 2.67 | 1.43 | 1.44 | 3.73 | 0.14 | 0.94 | <LOD | 7.1 |
| | | mean±SD | 0.39±NA | 1.85±0.79 | 3.25±2.15 | <LOD | 1.85±1.78 | 1.70±1.17 | 3.97±2.21 | 0.15±0.07 | 0.94±NA | 7.78±4.09 |
| >28 | 18 | median | 0.03 | 1.25 | 2.89 | 2.15 | 0.79 | 3.22 | <LOD | <LOD | <LOD | 6.81 |
| | | mean±SD | 0.03±NA | 1.15±0.40 | 3.01±1.27 | <LOD | 2.67±1.45 | 1.66±1.50 | 3.41±1.96 | <LOD | <LOD | 7.14±2.81 |
| **Gender** | | | | | | | | | | | | |
| male | 153 | median | 0.36 | 1.19 | 2.61 | 1.62 | 1.33 | 3.81 | 0.14 | 1.37 | <LOD | 7.25 |
| | | mean±SD | 0.40±0.32 | 1.54±0.78 | 3.20±2.17 | <LOD | 1.97±1.47 | 1.58±1.08 | 4.06±2.17 | 0.16±0.08 | 1.37±0.61 | 7.97±3.94 |
| female | 81 | median | 0.5 | 1.16 | 2.88 | 1.47 | 0.97 | 3.07 | 0.15 | <LOD | <LOD | 6.69 |
| | | mean±SD | 0.50±0.10 | 1.02±0.35 | 3.73±2.51 | <LOD | 1.63±0.91 | 1.12±0.71 | 3.50±1.83 | 0.18±0.10 | <LOD | 7.17±3.37 |

| Characteristic | | | ACE | CLO | DIN | IMI | IMID | NIT | THI | THIA | FLO | ΣNEOs |
|---|---|---|---|---|---|---|---|---|---|---|---|---|
| **Cancer** | | | | | | | | | | | | |
| yes | 125 | median | 0.39 | 1.19 | 2.6 | 1.45 | 1.23 | 3.8 | 0.13 | 1.37 | <LOD | 7.07 |
| | | mean±SD | 0.48±0.24 | 1.51±0.79 | 3.31±2.44 | <LOD | 1.65±0.97 | 1.45±0.89 | 3.97±2.15 | 0.16±0.09 | 1.37±0.61 | 7.60±3.65 |
| no | 109 | median | 0.3 | 1.18 | 2.89 | 1.65 | 1.15 | 3.23 | 0.18 | <LOD | <LOD | 6.94 |
| | | mean±SD | 0.30±0.38 | 1.09±0.42 | 3.48±2.12 | <LOD | 2.05±1.56 | 1.47±1.21 | 3.79±2.00 | 0.18±0.08 | <LOD | 7.82±3.93 |

Note: body mass index (BMI, kg/m2) = weight (kg)/height2 (m2); <LOD: lower than limit of detection (LOD); ∑NEOs: total concentration of eight individual neonicotinoids. Nine neonicotinoids including acetamiprid (ACE), thiamethoxam (THIA), imidacloprid (IMI), clothianidin (CLO), thiacloprid (THI), dinotefuran (DIN), nitenpyram (NIT), and imidaclothiz (IMID), flonicamid(FLO). The sample sizes of cancer and non-cancer groups in the subgroups (age, BMI, and gender) were also listed.

**Table S5. The neonicotinoid residues in the human liver samples for different demographic groups.**

| Characteristics | | | ACE | CLO | DIN | IMI | IMID | NIT | THI | THIA | FLO | ΣNEOs |
|---|---|---|---|---|---|---|---|---|---|---|---|---|
| **Detection frequency (DF, %)** | | | 3 | 5.6 | 84.6 | 40.6 | 32.1 | 59.4 | 14.1 | 0.9 | 0 | 84.6 |
| **Age，year** | **Sample size** | **Level** | | | | | ng/g | | | | | |
| 10-30 | 6 | median | 6.45 | <LOD | 10.26 | 3.91 | 2.3 | 8.19 | 4.87 | 6.84 | 0.39 | 32.98 |
| | | mean±SD | 6.37±1.96 | <LOD | 9.31±3.73 | 3.91±2.59 | 3.58±2.23 | 9.89±6.94 | 4.87±3.48 | 6.55±5.20 | 0.39±NA | 32.85±11.29 |
| 30-60 | 126 | median | 5.08 | 7.09 | 10.34 | 3.84 | 6.97 | 8.53 | 1.97 | 1.9 | 2.63 | 32.88 |
| | | mean±SD | 6.22±4.47 | 6.98±4.52 | 11.13±7.47 | 4.85±3.38 | 8.23±6.05 | 9.82±7.53 | 3.19±3.12 | 3.79±4.13 | 2.63±NA | 35.31±17.22 |
| 60-90 | 102 | median | 6.94 | 9.83 | 11.29 | 4.13 | 5.64 | 5.83 | 2.31 | 2.22 | 0.23 | 38.18 |
| | | mean±SD | 7.71±4.84 | 10.51±7.85 | 13.59±7.85 | 6.00±5.29 | 7.03±5.22 | 8.75±7.59 | 3.40±3.24 | 3.95±3.83 | 0.61±0.69 | 39.01±15.28 |
| **BMI，kg/m2** | | | | | | | | | | | | |
| <24 | 147 | median | 5.57 | 7.51 | 10.65 | 4.71 | 5.63 | 7.1 | 2.4 | 2.53 | 0.31 | 35.14 |
| | | mean±SD | 6.25±4.39 | 8.16±6.07 | 12.74±8.27 | 5.85±4.61 | 7.16±5.38 | 9.24±7.58 | 3.26±2.90 | 4.27±4.22 | 0.86±1.18 | 36.65±16.60 |
| 24-28 | 68 | median | 7.72 | 6.13 | 10.8 | 1.74 | 7.44 | 7.78 | 2.29 | 2.28 | 1.4 | 36.65 |
| | | mean±SD | 8.13±5.09 | 8.10±5.49 | 10.34±5.81 | 3.02±2.74 | 8.42±5.99 | 10.10±7.89 | 3.23±2.97 | 3.92±3.74 | 1.40±NA | 36.88±15.77 |
| >28 | 18 | median | 6.69 | 9.83 | 14.48 | 7.52 | 5.33 | 3.52 | 1.72 | 0.62 | <LOD | 44.66 |
| | | mean±SD | 6.88±4.21 | 11.09±9.33 | 14.72±7.61 | 7.52±3.62 | 7.93±6.52 | 6.33±5.25 | 3.68±5.20 | 1.09±1.61 | <LOD | 39.44±16.83 |
| **Gender** | | | | | | | | | | | | |
| male | 153 | median | 6.48 | 10.69 | 11.8 | 5.58 | 6.16 | 7.21 | 2.36 | 2.6 | 0.79 | 38.04 |
| | | mean±SD | 7.32±4.64 | 10.49±6.77 | 12.70±7.11 | 6.45±5.01 | 7.85±6.02 | 9.45±7.46 | 3.49±3.18 | 4.70±4.42 | 0.79±0.85 | 38.76±16.19 |
| female | 81 | median | 5.28 | 4.38 | 9.48 | 2.55 | 5.63 | 6.81 | 1.45 | 1.35 | 0.39 | 31.17 |
| | | mean±SD | 5.96±4.58 | 4.85±3.78 | 11.31±8.51 | 3.85±2.70 | 7.05±4.88 | 8.91±7.69 | 2.82±3.08 | 2.66±2.77 | 1.08±1.34 | 33.08±16.03 |

| Characteristics | | | ACE | CLO | DIN | IMI | IMID | NIT | THI | THIA | FLO | ΣNEOs |
|---|---|---|---|---|---|---|---|---|---|---|---|---|
| **Cancer** | | | | | | | | | | | | |
| yes | 125 | median | 6.14 | 9.81 | 11.38 | 3.52 | 6.13 | 7.01 | 2.17 | 2.64 | 0.89 | 37.97 |
| | | mean±SD | 7.02±4.63 | 9.93±6.21 | 13.05±7.43 | 5.14±4.78 | 7.52±5.44 | 9.78±7.95 | 3.48±3.45 | 4.30±3.85 | 1.16±1.11 | 38.40±16.83 |
| no | 109 | median | 6.05 | 4.87 | 10 | 4.97 | 6.45 | 7.19 | 2.52 | 1.28 | 0.19 | 32.53 |
| | | mean±SD | 6.68±4.71 | 7.10±6.66 | 11.04±7.83 | 5.61±3.88 | 7.66±5.98 | 8.62±6.91 | 3.04±2.74 | 3.44±4.18 | 0.19±NA | 34.81±15.46 |

Note: body mass index (BMI, kg/m2) = weight (kg)/height2 (m2); <LOD: lower than limit of detection (LOD); ∑NEOs: total concentration of eight individual neonicotinoids. Nine neonicotinoids including acetamiprid (ACE), thiamethoxam (THIA), imidacloprid (IMI), clothianidin (CLO), thiacloprid (THI), dinotefuran (DIN), nitenpyram (NIT), and imidaclothiz (IMID), flonicamid(FLO). The sample sizes of cancer and non-cancer groups in the subgroups (age, BMI, and gender) were also listed.

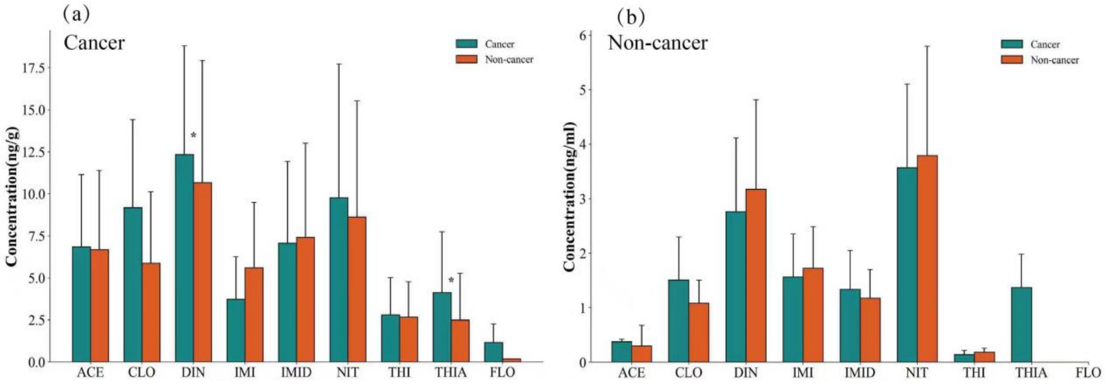


**Figure S1. Comparison of the mean concentrations of NEOs in liver tissue (a) and whole blood (b) between the liver cancer group and the non-liver cancer group.**

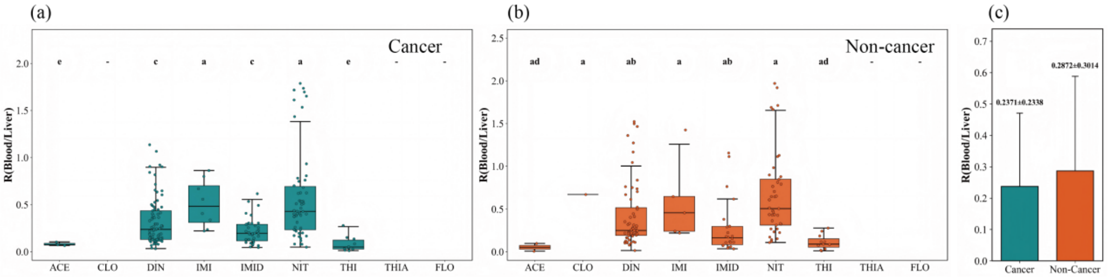


**Figure S2. Cancer (a) and non-cancer (b) populations distributions of R(Blood/Liver); (c) Comparison of R(Blood/Liver) for ∑NEOs between groups.**
